# Preoperative 3D FSE T1-Weighted MR Plaque Imaging for Severely Stenotic Cervical ICA: Accuracy of Predicting Emboli during Carotid Endarterectomy

**DOI:** 10.3390/ijms17111791

**Published:** 2016-10-27

**Authors:** Yasushi Ogasawara, Yuiko Sato, Shinsuke Narumi, Makoto Sasaki, Shunrou Fujiwara, Masakazu Kobayashi, Kenji Yoshida, Yasuo Terayama, Kuniaki Ogasawara

**Affiliations:** 1 Department of Neurosurgery, School of Medicine, Iwate Medical University, 19-1 Uchimaru, Morioka 020-8505, Japan; oneandonlytoearth@yahoo.co.jp (Y.O.); okiuyotas@yahoo.co.jp (Y.S.); shunfuji@iwate-med.ac.jp (S.F.); kobamasa@iwate-med.ac.jp (M.K.); kenyoshi@iwate-med.ac.jp (K.Y.); 2 Department of Neurology and Gerontology, School of Medicine, Iwate Medical University, 19-1 Uchimaru, Morioka 020-8505, Japan; snarumi@iwate-med.ac.jp (S.N.); teray@iwate-med.ac.jp (Y.T.); 3 Division of Ultra-High Field MRI and Department of Radiology, School of Medicine, Iwate Medical University, 19-1 Uchimaru, Morioka 020-8505, Japan; masasaki@iwate-med.ac.jp

**Keywords:** carotid endarterectomy, artery-to-artery embolism, plaque imaging, three-dimensional, fast spin echo magnetic resonance

## Abstract

The aim of the present study was to determine whether preoperative three-dimensional (3D) fast spin-echo (FSE) T1-weighted magnetic resonance (MR) plaque imaging for severely stenotic cervical carotid arteries could accurately predict the development of artery-to-artery emboli during exposure of the carotid arteries in carotid endarterectomy (CEA). Seventy-five patients underwent preoperative MR plaque imaging and CEA under transcranial Doppler ultrasonography of the ipsilateral middle cerebral artery. On reformatted axial MR image slices showing the maximum plaque occupation rate (POR) and maximum plaque intensity for each patient, the contrast ratio (CR) was calculated by dividing the internal carotid artery plaque signal intensity by the sternocleidomastoid muscle signal intensity. For all patients, the area under the receiver operating characteristic curve (AUC)—used to discriminate between the presence and absence of microembolic signals—was significantly greater for the CR on the axial image with maximum plaque intensity (CR_max intensity_) (0.941) than for that with the maximum POR (0.885) (*p* < 0.05). For 32 patients in whom both the maximum POR and the maximum plaque density were identified, the AUCs for the CR were 1.000. Preoperative 3D FSE T1-weighted MR plaque imaging accurately predicts the development of artery-to-artery emboli during exposure of the carotid arteries in CEA.

## 1. Introduction

Magnetic resonance (MR) plaque imaging is used to assess plaque characteristics in patients with cervical carotid stenosis. Of various two-dimensional (2D) T1-weighted sequences for MR plaque imaging, the spin echo (SE) T1-weighted imaging (WI) technique, with appropriate scanning parameters, is the most accurate for quantifying intraplaque components [1,2,3,4]. However, a three-dimensional (3D) fast spin-echo (FSE) T1-WI technique capable of minimizing partial volume effects and motion artifacts, enhancing black-blood effects, and maintaining T1-WI contrast has recently been adopted [5]. A comparison of diagnostic accuracy between 2D SE T1-WI and 3D FSE T1-WI for carotid plaque characterization using pathologic specimens excised from carotid endarterectomy (CEA) was performed. It was shown that, because it provides improved contrast of lipid-rich plaques, 3D FSE T1-WI could characterize carotid plaque composition more accurately than 2D SE T1-WI [5]. In addition, 3D FSE T1-WI could differentiate vulnerable from stable plaques with excellent sensitivity and specificity [5].

Although CEA has been shown to be an effective method for preventing stroke in select patients [6,7,8], more than 70% of intraoperative procedure-related strokes are due to cerebral embolism from the surgical site [9]. Intraoperative transcranial Doppler (TCD) monitoring of the middle cerebral artery (MCA) detects emboli from the surgical site as microembolic signals (MES) [9,10,11,12,13,14,15]. A significant correlation has been reported between detection of MES during exposure of the carotid arteries and the development of new ischemic lesions or neurological deficits after CEA [10,11,12,13,14,15]. When solid masses such as thrombi are present on the internal carotid artery (ICA) plaque surface, they can be dislodged by manipulating the carotid arteries for exposure during CEA, resulting in the development of cerebral ischemic lesions [16]. This suggests that MES during exposure of the carotid arteries may be associated with vulnerable carotid plaques [17]; therefore, identifying plaque vulnerability preoperatively may improve risk stratification in patients eligible for CEA.

A recent study demonstrated that, of various 2D T1-WI plaque imaging techniques, non-gated SE predicted the development of MES during carotid exposure in CEA more accurately than other MR plaque imaging techniques [18]. However, whereas the sensitivity and negative-predictive value for the 2D SE T1-WI in predicting the development of MES during carotid exposure were high, its specificity and positive-predictive value were less than 50%. In that study, the signal intensity of the carotid plaque was measured in only one axial section set at the location where the stenosis was most severe. This measurement may result in low specificity and positive-predictive value because MES do not always originate from the location with the most severe stenosis during exposure of the carotid arteries [18]. Therefore, analysis of plaque using 3D MR imaging may provide a more accurate prediction of the development of MES during carotid exposure.

The aims of the present study were (1) to determine whether preoperative 3D FSE T1-weighted plaque imaging for cervical carotid artery stenosis could accurately predict the development of MES on TCD during exposure of the carotid arteries in CEA; and (2) to compare the predictive accuracy of 3D FSE T1-weighted plaque imaging and 2D SE T1-weighted plaque imaging in historical controls [18].

## 2. Results

### 2.1. Clinical Characteristics

From December 2013 to August 2015, 88 patients satisfying the inclusion criteria consecutively underwent 3D FSE T1-WI and subsequent CEA, except for one who underwent urgent CEA because of crescendo transient ischemic attacks and thus did not undergo 3D FSE T1-WI. Although TCD was attempted in all participants, reliable TCD monitoring was not achieved throughout the entire operation in 12 patients because an adequate bone window could not be obtained. Therefore, after excluding these 13 patients from analysis, a total of 75 patients were enrolled into the present study. Table 1 shows the basic characteristics of these 75 patients and comparisons with 80 patients as historical controls who were consecutively measured using 2D SE T1-WI from July 2010 to January 2012 [18]. No significant differences were found in background characteristics between the two groups.

### 2.2. Intraoperative and Postoperative Events

When the ICA was manipulated to allow exposure from the carotid sheath, MES were detected in 19 (25%) of the 75 patients. Among these 19 patients, two developed neurological deficits after recovery from general anesthesia; all deficits included hemiparesis contralateral to the CEA and completely resolved within 12 h. None of the remaining 56 patients (without MES during exposure of the carotid arteries) experienced postoperative neurological deficits.

### 2.3. Relationship between the Image with the Maximum Plaque Occupation Rate and the Image with the Maximum Plaque Intensity

On 3D FSE T1-WI, it was determined that both the image with the maximum plaque occupation rate (POR_max occupation_) and the image with the maximum plaque intensity were identified in 32 patients (43%). In the remaining 43 patients (57%), the distance between the two images ranged from 3 to 17 mm (6 ± 4 mm).

### 2.4. Relationship between Development of Microembolic Signals (MES) during Exposure of the Carotid Arteries and the Plaque Occupation Rate or the Plaque Intensity

**Figure 1:**
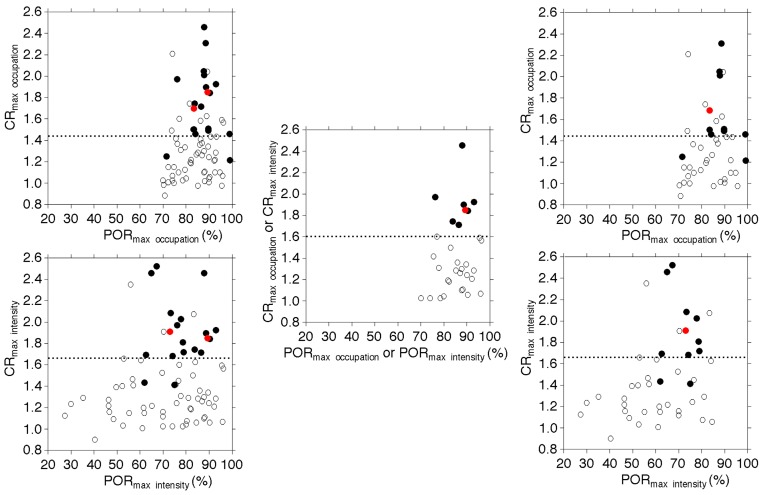
Relationship between the plaque occupation rate (POR) in the image with the maximum POR (POR_max occupation_) or that in the image with the maximum plaque intensity (POR_max intensity_), contrast ratio (CR) in the image with POR_max occupation_ (CR_max occupation_), or that in the image with the maximum plaque intensity (CR_max intensity_) and development of microembolic signals (MES) during exposure of the carotid arteries (**left**, for all patients; **middle**, for patients with identification of the image with the POR_max occupation_ and the image with the maximum plaque intensity; **right**, for patients without this identification). In the **middle** panel, each POR_max occupation_ or CR_max occupation_ is identical to POR_max intensity_ or CR_max intensity_, respectively. Closed and open circles indicate patients with and without MES, respectively. Red and black circles indicate patients with and without postoperative new neurological deficits, respectively. Dashed horizontal lines denote the cutoff points closest to the **left upper** corners of the receiver operating characteristic (ROC) curves in predicting the development of MES.

Figure 1 shows the relationship between POR_max occupation_, POR in the image with the maximum plaque intensity (POR_max intensity_), contrast ratio (CR) on the image with the POR_max occupation_ (CR_max occupation_), or CR on the image with the maximum plaque intensity (CR_max intensity_) and development of MES during exposure of the carotid arteries.

Table 2 shows the areas under the receiver operating characteristic curves (AUCs) and the sensitivity, specificity, and positive- and negative-predictive values for CR_max occupation_ and CR_max intensity_ at the cutoff point closest to the left upper corner of the receiving operator characteristic (ROC) curve for predicting the development of MES during exposure of the carotid arteries.

**Figure 2:**
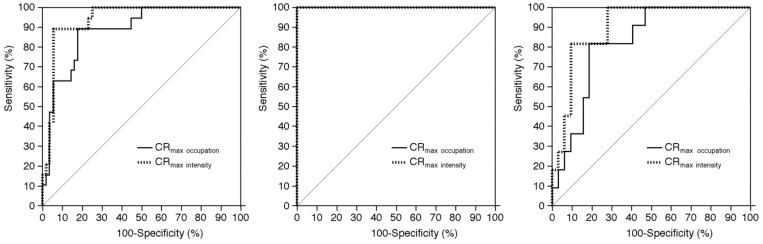
Receiving operator characteristic (ROC) curves used to compare predictive accuracy between CR_max occupation_ and CR_max intensity_ for the development of MES during exposure of the carotid arteries (**left**, for all patients; **middle**, for patients with identification of the image with the POR_max occupation_ and that with the maximum plaque intensity; **right**, for patients without this identification). In the **middle** panel, each area under the ROC curve for CR_max occupation_ or CR_max intensity_ (each CR_max occupation_ is identical to CR_max intensity_, respectively) is 1.000.

Figure 2 shows the ROC curves for CR_max occupation_ and CR_max intensity_ in predicting the development of MES during exposure of the carotid arteries.

For all patients, the AUC for CR_max intensity_ was significantly greater than that for CR_max occupation_. For patients with identification of the image with both the POR_max occupation_ and the maximum plaque intensity, the AUCs for CR_max occupation_ and CR_max intensity_ were 1.000. For patients without this identification, the AUC was significantly greater for CR_max intensity_ than for CR_max occupation_. Furthermore, while the AUCs for CR_max intensity_ did not differ between patients with and without the identification, the AUCs for CR_max occupation_ were significantly greater for the former than the latter.

Of two patients with postoperative neurological deficits, one patient had identification of the image with both the POR_max occupation_ and the maximum plaque intensity; CR_max occupation_ (=CR_max intensity_) was 1.85 (Figure 1). Another patient with the deficit had no identification of the two images; CR_max occupation_ and CR_max intensity_ were 1.69 and 1.91, respectively (Figure 1). These values were greater than each cutoff point closest to the left upper corner of the ROC curve for predicting the development of MES during exposure of the carotid arteries.

Sixteen patients with CR_max occupation_ ≥ 1.60 were classified into two subgroups: eight patients with higher CR_max occupation_ (≥1.90) and eight patients with lower CR_max occupation_ (between 1.60 and 1.90). The incidence of MES during exposure of the carotid arteries did not differ between these two subgroups (6/8 (75%) for higher CR_max occupation_; 6/8 (75%) for lower CR_max occupation_) (*p* > 0.9999). Twenty-three patients with CR_max intensity_ ≥ 1.60 were classified into two subgroups: 11 patients with higher CR_max intensity_ (≥1.90) and 12 patients with lower CR_max intensity_ (between 1.60 and 1.90). The incidence of MES during exposure of the carotid arteries did not differ between these two subgroups (8/11 (73%) for higher CR_max intensity_; 9/12 (75%) for lower CR_max intensity_) (*p* > 0.9999).

The results of univariate analysis of factors related to the development of MES during exposure of the carotid arteries (except CR_max occupation_, CR_max intensity_, POR_max occupation_, and POR_max intensity_) are summarized in Table 3.

Patients with MES had a significantly higher prevalence of symptomatic lesions and ulceration of stenotic lesion than those without. No other significant associations with the development of MES during exposure of the carotid arteries were observed. In multivariate analysis of factors related to the development of MES during exposure of the carotid arteries, symptomatic lesions and ulceration of stenotic lesion, as items showing *p* < 0.2 in univariate analyses, were used as confounders in the logistic regression model. The AUC in predicting the development of MES during exposure of the carotid arteries was greater for CR_max intensity_ than for CR_max occupation_, and POR_max occupation_ was also added as a confounder. As a result, only CR_max intensity_ was significantly associated with the development of MES during exposure of the carotid arteries (95% confidence interval (CI): 17.5–351.5; *p* < 0.0001).

**Figure 3:**
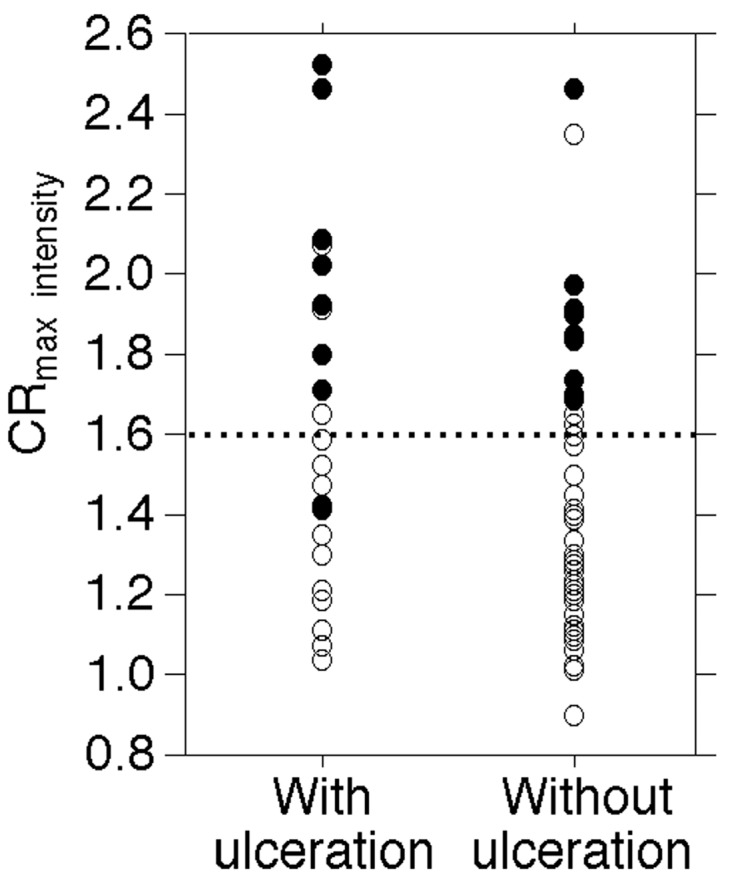
Relationship among ulceration of stenotic lesions, CR_max intensity_, and development of MES during exposure of the carotid arteries. Closed and open circles indicate patients with and without MES, respectively. Dashed horizontal line denotes CR_max intensity_ of 1.60.

Figure 3 shows the relationship among ulceration of stenotic lesions, CR_max intensity_ and development of MES during exposure of the carotid arteries.

In a subgroup of patients with CR_max intensity_ < 1.60, the incidence of the MES was greater in patients with ulceration (2/12 (17%) than in those without (0/40 (0%)) (*p* = 0.0498); in another subgroup of patients with CR_max intensity_ ≥ 1.60, the incidence did not differ between patients with (8/11 (73%)) and without (9/12 (75%)) the ulceration (*p* > 0.9999).

### 2.5. Comparisons of Area under Curve (AUC), Sensitivity, Specificity, and Positive- and Negative-Predictive Values for CR_max occupation_ or CR_max intensity_ and Those for CR in Historical Controls Measured Using 2D SE T1-WI

No differences were observed between the AUC of CR_max occupation_ or CR_max intensity_ or that of CR in the historical controls (Table 2). While sensitivity and positive- and negative-predictive values did not differ between CR_max occupation_ and CR in the historical controls, specificity was significantly greater for the former than for the latter (Table 2). While the sensitivity and negative-predictive value did not differ between CR_max intensity_ and CR in the historical controls, the specificity and positive-predictive value were significantly greater for the former than for the latter (Table 2).

## 3. Discussion

### 3.1. Findings

The results of the present study demonstrated that preoperative 3D FSE T1-weighted MR plaque imaging for cervical carotid artery stenosis could accurately predict the development of MES on TCD during exposure of the carotid arteries in CEA. 3D FSE T1-WI plaque imaging may therefore provide greater predictive accuracy than 2D SE T1-weighted plaque imaging.

### 3.2. Data Interpretation

3D MR plaque imaging minimizes partial volume effects and motion artifacts, enhances black-blood effects, and maintains T1-WI contrast [1,2,3,4]. Furthermore, 2D images can be obtained in arbitrary sections from 3D imaging data. In the present study, 2D axial images perpendicular to the long axis of the common carotid artery and the ICA were generated from 3D imaging data. These methods may be suitable for the assessment of carotid plaques because the carotid artery is tortuous and the plaques are typically elongated in a superoinferior direction [5]. While several investigators have measured plaque intensity on the axial image slice with the greatest degree of carotid stenosis [1,3,4,18,19], others have measured plaque intensity on the image slice with the highest plaque intensity [20,21,22]. However, it remains unclear which of these two methods is more suitable for predicting the development of new postoperative ischemic events in patients undergoing carotid artery stenting or CEA or the development of future ischemic events in patients treated with medication alone. Therefore, we evaluated plaque intensity on two axial images obtained from 3D images as follows: the image slice with the maximum POR—possibly corresponding to the image slice with the greatest degree of carotid stenosis on angiography—and the image slice with the maximum plaque intensity. As a result, plaque intensity on the latter image more accurately predicted the development of MES during exposure of the carotid arteries than that on the former image. Furthermore, these two images failed to be identified in more than half of our patients, and plaque intensity more accurately predicted the development of MES for patients with identification than for those without. In addition, while the sensitivity and negative-predictive value in predicting the development of MES during carotid exposure did not differ between 3D FSE T1-WI and 2D SE T1-WI, the specificity and positive-predictive value were significantly greater in the former than in the latter. These findings suggest that 3D plaque-imaging is effective for predicting the development of MES in patients undergoing CEA for carotid stenosis; the findings also support the hypothesis that MES do not always originate from the site with the most severe stenosis during exposure of the carotid arteries.

According to a study that used the same methods for performing MR plaque imaging and measuring plaque intensity as those used in the present study [5], CR in 3D FSE T1-WI identified intraplaque components with a sensitivity and specificity of >90%: CRs in calcified lesion or fibrotic plaque without a lipid core ranged from 0.94 to 1.29; CRs in lipid-rich or necrotic core ranged from 1.33 to 1.54; and CRs in plaque with hemorrhage or thrombus were greater than 1.53. In particular, plaque with a CR ≥ 1.60 always indicated hemorrhage or thrombus. Our data showed that the optimal cutoff point for the CR on the image with the maximum plaque intensity was 1.66 or 1.60. These data correspond to previous findings that the development of MES during exposure of the carotid arteries in CEA is strongly associated with carotid plaque that histopathologically consists of hemorrhage [17]. Intraplaque hemorrhage depicted on MR plaque imaging is reportedly related to a histologically disrupted plaque surface (fissured fibrous cap), implying that thrombi are exposed to blood flow in patients with severe carotid artery stenosis (>70%) [23]. Surgical manipulation of the carotid arteries with such plaques likely leads to the development of emboli from this vulnerable plaque.

Several investigators have histopathologically classified intraplaque hemorrhage in the cervical carotid arteries into three stages: fresh (<1 week after hemorrhage, intact red blood cells with intracellular methemoglobin), recent (1–6 weeks after hemorrhage, lytic red blood cells with extracellular methemoglobin), and old (>6 weeks after hemorrhage, amorphous material) [24,25]. They also correlated these histological categories with findings on MR plaque imaging [24,25]. Fresh and recent intraplaque hemorrhages exhibited hyperintensity on T1-WI MR, and the former signal intensity was stronger than the latter [24,25]. Thus, while a plaque with a CR ≥ 1.60 consists of hemorrhage or thrombus, a plaque containing fresh hemorrhage may more greatly exhibit higher CR on MR plaque imaging. We also developed a hypothesis that when the carotid arteries are surgically manipulated, embolism from the surgical site is more likely to develop in the carotid arteries with fresh intraplaque hemorrhage than in those with recent intraplaque hemorrhage. To validate this hypothesis, we compared the incidence of MES during exposure of the carotid arteries between patients with higher CRs and lower CRs, but only in patients with a CR ≥ 1.60. We found that CR was not associated with a tendency to develop MES during exposure of the carotid arteries among patients with CR ≥ 1.60.

In the present study, although patients with MES during exposure of the carotid arteries had a significantly higher prevalence of ulceration of a stenotic lesion than those without, only CR_max intensity_ was significantly associated with the development of MES using the logistic regression model. In further analyses, development of MES was related to the presence of ulceration of a stenotic lesion in patients with CR_max intensity_ < 1.60, which indicates plaque composed of components other than hemorrhage or thrombus. However, this relationship does not exist in patients with CR_max intensity_ ≥ 1.60, which indicates plaque composed of hemorrhage or thrombus. This may be a reason why ulceration of a stenotic lesion was a weaker predictor of development of MES than CR_max intensity_.

### 3.3. Clinical Applications

Caplan and Hennerici [26] have reported that hemodynamic and embolic mechanisms are strictly linked and may interact to determine the ultimate degree of cerebral ischemia. According to their concept, low blood flow velocity in the cerebral artery may impair clearance of emboli generated from a proximal lesion, subsequently facilitating the onset of ischemia caused by emboli in poorly perfused areas of the brain. Indeed, low blood-flow velocity in the MCA reportedly correlated with the development of diffusion-weighted imaging (DWI)-characterized postoperative cerebral ischemic lesions related to the generation of microemboli during exposure of the carotid arteries in CEA [27]. The concept presented by Caplan and Hennerici also suggests that increased blood-flow velocity may prevent the development of cerebral ischemic lesions due to emboli. Actually, another study showed that the incidence of DWI-characterized postoperative cerebral ischemic lesions was significantly lower in patients with MCA blood-flow velocity increased by intentional hypertension during exposure of the carotid arteries than in patients without such a procedure [28]. Thus, we attempted to keep the increase in systolic blood pressure at least 10% above the preoperative value during exposure of the carotid arteries.

On the basis of these findings and our data, we propose the following practical clinical algorithm to prevent development of MES-related ischemic events in CEA. Patients undergo preoperative MR imaging of plaque using 3D FSE T1-WI; when the CR_max intensity_ is >1.60 for patients with identification of the image with both the POR_max occupation_, and the maximum plaque intensity or the CR_max intensity_ is >1.66 for patients without such identification, intentional hypertension is performed during exposure of the carotid arteries; and for other combinations, intentional hypertension is unnecessary.

### 3.4. Study Limitations

The present study did have several limitations. First, we did not directly compare the predictive accuracy of 3D FSE T1-WI with that of 2D SE T1-WI in identical subjects. Second, to standardize the surgical procedures as much as possible, all surgeries were performed by the same senior neurosurgeon, who was blinded to the intraoperative TCD findings and therefore continued with the surgeries regardless of what was found. However, the degree of stress on the carotid arteries due to manipulation during exposure might not have been equivalent among all patients, and this may have affected the development of MES. Finally, the small sample size and patient selection bias (14% of patients were excluded because of failure to obtain TCD data) were also limitations.

## 4. Materials and Methods

### 4.1. Subjects

The present study was designed as a prospective observational study, and the case cohort was compared with historical controls [18] composed of patients who underwent preoperative 2D SE T1-weighted plaque imaging and CEA. This study was approved by the Regional Ethical Board of Iwate Medical University (H22-31) and conducted in compliance with the Helsinki Declaration. Written, informed consent was obtained from all patients or their next of kin prior to participation.

The present study included patients with ipsilateral ICA stenosis ≥70% as per the North American Symptomatic Carotid Endarterectomy Trial [8]. Patients underwent the previously described angiography study [18] with arterial catheterization, had useful residual function (modified Rankin disability scale, 0–2), and underwent CEA of the carotid bifurcation in our institution. Patients who did not undergo preoperative 3D FSE T1-WI were excluded, as were patients in whom reliable TCD monitoring could not be obtained throughout the surgery due to a failure to obtain an adequate bone window.

### 4.2. Preoperative, Intraoperative, and Postoperative Management

Before surgery, on angiography with arterial catheterization, the length of the ICA stenotic lesion was measured, and the height of the distal end of the lesion relative to the cervical vertebra was determined in the lateral view; lesion tortuosity was defined using a previously described method [18]; and according to the method presented by Randoux et al. [18,29], a stenotic lesion was classified as having ulceration when it fulfilled the radiographic criteria for an ulcer niche, seen in profile as a crater penetrating a stenotic lesion in any projection.

Antiplatelet therapy was administered to all patients until the morning of the day on which CEA was performed. Furthermore, all patients underwent surgery under general anesthesia with an operative microscope. All skin incisions were made by the same senior neurosurgeon who was blinded to the MR plaque imaging findings. Dissection of the carotid sheath and exposure of the carotid arteries were routinely performed as follows [18,30]. First, the upper plane of the carotid sheath surrounding the common carotid artery was cut with scissors; next, the other planes of the carotid sheath were bluntly separated from the common and external carotid arteries and the ICA with Pean forceps. Scissors were used when the carotid sheath was adhered to the carotid arteries. The surgeon was blinded to the intraoperative TCD findings and proceeded with surgical procedures regardless of these findings.

During exposure of the carotid arteries, attempts were made to keep the increase in systolic blood pressure at least 10% above the preoperative value [28]. If needed, a vasodilator (nitroglycerin or nicardipine) or a vasoconstrictor (theoadrenalin) was administered intravenously. No intraluminal shunt or patch graft was used in these procedures [18,28]. Prior to ICA clamping, a bolus of heparin (5000 IU) was administered.

All patients were neurologically tested immediately before the induction of and after recovery from general anesthesia by a neurologist who was blinded to the patients’ clinical information, and the presence or absence of new postoperative neurological deficits was recorded [18].

### 4.3. Magnetic Resonance (MR) Plaque Imaging and Data Processing

Preoperative sagittal 3D FSE T1-WI of the affected carotid bifurcation was performed within 1 week prior to CEA using a 1.5-T MR imaging scanner (Signa HDxt; GE Healthcare, Milwaukee, WI, USA) and an eight-channel neurovascular coil under a previously described imaging protocol [5]. The voxel size was 0.5 m × 0.5 m × 0.5 m.

**Figure 4:**
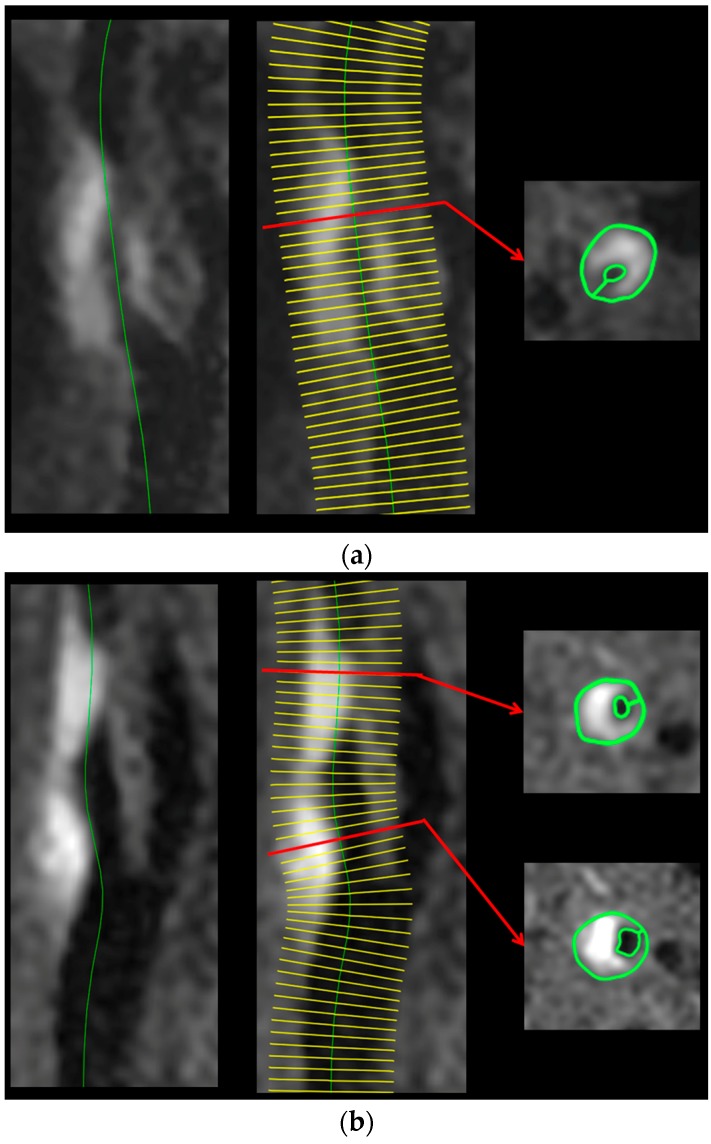
Preoperative three-dimensional fast spin echo T1-weighted magnetic resonance (3D FSE T1-weighted MR) plaque images of two patients with MES during exposure of the carotid arteries in endarterectomy. (**a**) The curved planar reformation is performed parallel to the long axis of the common and internal carotid arteries (**left**). The green line indicates the center line of the vessel lumen. On the image of the curved planar reformation, axial images with 1.0 mm thickness are reformatted as sections perpendicular to the center line (**middle**). Yellow lines indicate the position of each reformatted axial image. A reformatted axial image with the maximum POR and maximum plaque intensity (red arrows) are determined by tracing a plaque and vessel lumen of the common or internal carotid artery (**right**, green lines). A 65-year-old man with asymptomatic right internal carotid artery stenosis exhibits identification of the image with the maximum POR and the image with the maximum plaque intensity; (**b**) Preoperative 3D FSE T1-weighted MR plaque images of two patients with MES during exposure of the carotid arteries in endarterectomy. The curved planar reformation is performed parallel to the long axis of the common and internal carotid arteries (**left**). The green line indicates the center line of the vessel lumen. On the image of the curved planar reformation, axial images with 1.0 mm thickness are reformatted as sections perpendicular to the center line (**middle**). Yellow lines indicate the position of each reformatted axial image. A reformatted axial image with the maximum POR and maximum plaque intensity (red arrows) are determined by tracing a plaque and vessel lumen of the common or internal carotid artery (**right**, green lines). A 77-year-old man with symptomatic right internal carotid artery stenosis exhibits no identification of the image with the maximum POR (**upper**) and the image with the maximum plaque intensity (**lower**).

An investigator (blinded to other data) processed the 3D FSE T1-WI data using a free software package (OsiriX; Pixmeo, Geneva, Switzerland) as follows (Figure 4). The curved planar reformation image was generated parallel to the long axis of the common carotid artery and the ICA by manually setting and automatically connecting reference points in the center of the vessel lumen on each axial source image. On the curved planar reformation image with the center line, axial images with 1.0 mm thickness were newly reformatted as sections perpendicular to the center line. Thus, the final voxel size was 0.5 m × 0.5 m × 1.0 m.

In each reformatted axial image, the investigator manually traced a plaque and vessel lumen of the common carotid artery or the ICA (Figure 4). The resulting area was obtained in each image. First, POR was defined as follows: (an area of a plaque divided by an area of a vessel lumen) ×100 (%). In each patient, the image with the maximum value of the POR (defined as POR_max occupation_) was determined. Next, a signal intensity of the traced plaque was obtained on each image (total of 51 images) between 25 mm above and below the image with the POR_max occupation_ in each patient. Of these 51 images, the image with the highest value of the signal intensity was determined and defined as the image with the maximum plaque intensity. In the image with the maximum plaque intensity, the POR was also determined (defined as POR_max intensity_). In each patient, when the distance between the image with the POR_max occupation_ and the image with the maximum plaque intensity ranged from 0–2 mm, the patient was defined as having identification of the two images (Figure 4).

Of the 51 images in each patient, the image on which the sternocleidomastoid muscle adjacent to the carotid arteries was displayed as larger was determined, after which the investigator manually traced the muscle and measured the signal intensity. The CR on the image with the POR_max occupation_ (defined as CR_max occupation_) and that on the image with the maximum plaque intensity (defined as CR_max intensity_) were calculated by dividing the signal intensity of the plaque by that of the muscle.

### 4.4. Transcranial Doppler (TCD) Monitoring

TCD was performed using a PIONEER TC2020 system (EME, Uberlingen, Germany; software version 2.50, 2-MHz probe; diameter, 1.5 cm; insonation depth, 40–66 mm; scale, −100 and +150 cm/s; sample volume, 2 mm; 64-point fast Fourier transform; fast Fourier transform length, 2 mm, fast Fourier transform overlap, 60%; high-pass filter, 100 Hz; detection threshold, 9 dB; minimum increase time, 10 ms) for insonation of the MCA ipsilateral to the carotid artery undergoing CEA [18]. TCD data were stored on a hard disk using a coding system and were later manually analyzed by a clinical neurophysiologist who was blinded to patient information [18]. MES were identified during exposure of the carotid arteries (from skin incision until ICA clamping) according to the recommended guidelines [31].

### 4.5. Statistical Analysis

Data are expressed as the mean ± standard deviation (SD). Differences of variables between two groups were evaluated using the Mann–Whitney U test or the χ^2^ test. The accuracy of the CR to predict development of MES during exposure of the carotid arteries was determined using an ROC curve, and the ability to discriminate between the presence or absence of MES during exposure of the carotid arteries was estimated using the AUC. Pairwise comparison of the AUCs using the method proposed by Pepe and Longton [32] was performed between CR_max occupation_ and CR_max intensity_. Differences in the AUCs for the CR between patients with and without identification of the image with the POR_max occupation_ and that with the maximum plaque intensity were analyzed using 95% CIs. Differences in the AUC, sensitivity, specificity, and positive- and negative-predictive values between the CR_max occupation_ or CR_max intensity_ and CR in historical controls [18] that were measured using 2D SE T1-WI at the location at which the stenosis was most severe were also analyzed using 95% CIs. The relationship between development of MES during exposure of the carotid arteries and each variable (except CR and POR) was evaluated with univariate analysis using the Mann–Whitney U test or the χ^2^ test. Multivariate statistical analysis of factors related to the development of MES during exposure of the carotid arteries was performed using logistic regression modeling. Variables showing values of *p* < 0.2 in univariate analyses were entered into the final model. For all statistical analyses, significance was set at the *p* < 0.05 level.

## 5. Conclusions

The results of the present study demonstrated that preoperative 3D FSE T1-weighted plaque imaging for cervical carotid artery stenosis accurately predicts the development of MES on TCD during exposure of the carotid arteries in CEA. These findings also suggest that 3D FSE T1-weighted plaque imaging may provide greater predictive accuracy than 2D SE T1-weighted plaque imaging.

## Figures and Tables

**Table 1:** Comparison of basic characteristics between patients undergoing 3D fast spin-echo (FSE) T1-weighted imaging (WI) plaque imaging and historical controls undergoing 2D T1-WI SE plaque imaging. ICA, internal carotid artery; SD, standard deviation.

Patient Characteristics	3D FSE T1-WI Plaque Imaging (*n* = 75)	Historical Controls (*n* = 80) [18]	*p*
Age (years) (mean ± SD)	69.8 ± 7.0	69.4 ± 6.8	0.6231
Male sex	68 (91%)	77 (96%)	0.2762
Hypertension	68 (91%)	64 (80%)	0.0728
Diabetes mellitus	26 (35%)	26 (33%)	0.8651
Dyslipidemia	24 (32%)	21 (26%)	0.4810
Symptomatic lesions	47 (62%)	50 (63%)	0.9998
Degree of ICA stenosis (%) (mean ± SD)	87.6 ± 8.8	88.2 ± 8.4	0.8056
Length of stenotic lesion (mm) (mean ± SD)	54.3 ± 10.8	53.7 ± 11.7	0.6482
Height of distal end of stenotic lesion relative to cervical vertebra (mean ± SD) (mmHg) (mean ± SD)	2.7 ± 0.9	2.8 ± 0.8	0.8421
Tortuosity of stenotic lesion (°) (mean ± SD)	111.4 ± 25.8	109.9 ± 23.8	0.7838
Ulceration of stenotic lesion	23 (31%)	27 (34%)	0.7327

**Table 2:** Area under the ROC curve (AUC), sensitivity, specificity, and positive- and negative-predictive values for contrast ratio (CR) in the development of microembolic signals (MES) during exposure of the carotid arteries. NS, not significant.

Items	All Patients (*n* = 75)		Patients with Identificatio*n* of the Two Images * (*n* = 32)	Patients without Identification of the Two Images * (*n* = 43)		*F* CR in Historical Controls (*n* = 80) [18]	*p*					
							*A* vs. *B*	*D* vs. *E*	*C* vs. *D*	*C* vs. *E*	*A* vs. *F*	*B* vs. *F*
	*A* CR_max occupation_	*B* CR_max intensity_	*C* CR_max occupation_ or CR_max intensity_	*D* CR_max occupation_	*E* CR_max intensity_							
AUC	0.885	0.941	1.000	0.824	0.901	0.821	<0.05 **	<0.01 ***	<0.05	NS	NS	NS
95% CI	0.791–0.947	0.861–0.982	0.925–1.000	0.677–0.923	0.770–0.971	0.723–0.901						
Sensitivity	90%	90%	100%	82%	82%	100%	-	-	-	-	NS	NS
95% CI	67%–99%	67%–99%	82%–100%	48%–98%	48%–98%	85%–100%						
Specificity	82%	95%	100%	81%	91%	49%	-	-	-	-	<0.05	<0.05
95% CI	67%–91%	85%–99%	88%–100%	67%–93%	75%–98%	36%–63%						
Positive predictive value	63%	85%	100%	60%	75%	44%	-	-	-	-	NS	<0.05
95% CI	42%–81%	62%–97%	82%–100%	32%–80%	43%–95%	31%–59%						
Negative predictive value	96%	96%	100%	93%	94%	100%	-	-	-	-	NS	NS
95% CI	88%–100%	88%–100%	88%–100%	77%–99%	79%–99%	88%–100%						
Cutoff point	1.44	1.66	1.60	1.44	1.66	1.16	-	-	-	-	-	-

* The image with the maximum plaque occupation rate and the image with the maximum plaque intensity; ** difference between AUCs of 0.056, pairwise comparison; *** difference between AUCs of 0.077, pairwise comparison.

**Table 3:** Risk factors related to the development of MES during exposure of the carotid arteries.

Factors	Development of MES		*p*
	Yes (*n* = 19)	No (*n* = 56)	
Age (years) (mean ± SD)	69.6 ± 7.8	69.9 ± 6.8	0.8119
Male sex	18 (95%)	50 (89%)	0.6709
Hypertension	18 (95%)	50 (89%)	0.6709
Diabetes mellitus	9 (47%)	17 (30%)	0.2641
Dyslipidemia	5 (26%)	19 (34%)	0.7765
Symptomatic lesion	16 (84%)	31 (55%)	0.0296
Degree of ICA stenosis (%) (mean ± SD)	85.9 ± 9.6	87.9 ± 8.7	0.5011
Length of stenotic lesion (mm) (mean ± SD)	53.6 ± 11.8	55.6 ± 10.1	0.5143
Height of distal end of stenotic lesion relative to cervical vertebra (mean ± SD)	2.8 ± 1.1	2.7 ± 0.8	0.7432
Tortuosity of stenotic lesion (°) (mean ± SD)	109.3 ± 27.2	112.0 ± 24.9	0.4328
Ulceration of stenotic lesion	10 (53%)	13 (23%)	0.0226
Identification of an image with POR_max occupation_ and that with maximal plaque intensity	8 (42%)	24 (43%)	>0.9999

## Abbreviations

MR	magnetic resonance
MCA	middle cerebral artery
3D FSE T1-WI	three-dimensional fast spin echo T1-weighted imaging
2D SE T1-WI	two-dimensional spin echo T1-weighted imaging
SD	standard deviation
ICA	internal carotid artery
CEA	carotid endarterectomy
TCD	transcranial Doppler
MES	microembolic signals
POR_max occupation_	maximum plaque occupation rate
AUC	area under receiver operating characteristic curve
CR	contrast ratio
CI	confidence interval
vs.	versus
